# Electron microscopy reveals water networks inside hydrated cellulose fibers

**DOI:** 10.64898/2026.09.16.752152

**Published:** 2026-09-29

**Authors:** Ruoya Ho, Arunabh Athreya, Yanina Pankratova, Chaemyeong Lim, Louis Wilson, Mei Hong, Keisuke Nakashima, Jochen Zimmer

## Abstract

Cellulose is a highly abundant linear glucose polymer that exists in amorphous and fibrillar forms ^1-3^ . Primarily produced by vascular plants but also microbes and tunicates, cellulose predominantly performs architectural functions by stabilizing cell walls, tissues, and multicellular communities ^4-9^ . Owing to its association with other cell surface materials, physiological higher-order structures of cellulose have been difficult to obtain. Here, we describe a never-dried tunicate cellulose fibril structure by cryogenic electron microscopy, which resolves a fiber of more than 360 cellulose strands. Our data reveals linear and severely twisted fiber segments that are interspersed with solvent channels. Solid-state NMR analyses and molecular dynamics simulations indicate the presence of structural water molecules that form a network connecting neighboring cellulose chains. Confocal and super-resolution MINFLUX fluorescence imaging using cellulose-specific probes as well as biochemical analyses demonstrate shared surface and material properties of plant and tunicate cellulose fibers. Combined, our data suggest that architectural water molecules may be common features of cellulose fibers and likely other polysaccharide assemblies in their hydrated physiological states.

## Full Text Availability

The license terms selected by the author(s) for this preprint version do not permit archiving in PMC. The full text is available from the preprint server.

